# Active microbial ecosystem in glacier basal ice fuelled by iron and silicate comminution‐derived hydrogen

**DOI:** 10.1002/mbo3.1200

**Published:** 2021-07-19

**Authors:** Mario Toubes‐Rodrigo, Sanja Potgieter‐Vermaak, Robin Sen, Edda S. Oddsdóttir, David Elliott, Simon Cook

**Affiliations:** ^1^ AstrobiologyOU Faculty of Science, Technology, Engineering and Mathematics The Open University Milton Keynes UK; ^2^ Department of Natural Sciences Ecology and Environment Research Centre Manchester Metropolitan University Manchester UK; ^3^ Icelandic Forest Research Reykjavik Iceland; ^4^ Environmental Sustainability Research Centre University of Derby Derby UK; ^5^ Geography and Environmental Science University of Dundee Dundee UK; ^6^ UNESCO Centre for Water Law, Policy and Science University of Dundee Dundee UK

**Keywords:** cryosphere, environmental microbiology, extremophiles, glaciers, microbial ecology

## Abstract

The basal zone of glaciers is characterized by physicochemical properties that are distinct from firnified ice due to strong interactions with underlying substrate and bedrock. Basal ice (BI) ecology and the roles that the microbiota play in biogeochemical cycling, weathering, and proglacial soil formation remain poorly described. We report on basal ice geochemistry, bacterial diversity (16S rRNA gene phylogeny), and inferred ecological roles at three temperate Icelandic glaciers. We sampled three physically distinct basal ice facies (stratified, dispersed, and debris bands) and found facies dependent on biological similarities and differences; basal ice character is therefore an important sampling consideration in future studies. Based on a high abundance of silicates and Fe‐containing minerals and, compared to earlier BI literature, total C was detected that could sustain the basal ice ecosystem. It was hypothesized that C‐fixing chemolithotrophic bacteria, especially Fe‐oxidisers and hydrogenotrophs, mutualistically support associated heterotrophic communities. Basal ice‐derived rRNA gene sequences corresponding to genera known to harbor hydrogenotrophic methanogens suggest that silicate comminution‐derived hydrogen can also be utilized for methanogenesis. PICRUSt‐predicted metabolism suggests that methane metabolism and C‐fixation pathways could be highly relevant in BI, indicating the importance of these metabolic routes. The nutrients and microbial communities release from melting basal ice may play an important role in promoting pioneering communities establishment and soil development in deglaciating forelands.

## Graphical abstract

The subglacial ecosystem is globally significant because glaciers and ice sheets cover ~10% of the Earth's land surface, yet it remains poorly understood. This study provides a detailed characterization of the geochemistry and bacterial community inhabiting the subglacial basal ice layer of three Icelandic glaciers. Our data show that a thriving chemolithotroph‐powered community exists within basal ice, fuelled by the oxidation of hydrogen and iron.

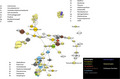

## 1 INTRODUCTION

Glaciers represent important ecosystems (e.g., (Anesio et al., 2009; Hodson et al., 2008; Hotaling et al., 2017)). Glacial microbiology research has mostly been focused on understanding the supraglacial ecosystem, in large part because of its relevance to albedo, and hence the surface mass balance of glaciers and ice sheets (Bradley et al., 2016; Edwards, Douglas, et al., 2013; Edwards, Rassner, et al., 2013; Kaczmarek et al., 2016; Lutz et al., 2014, 2015). The subglacial environment has received much less attention but is thought to be important for bedrock‐derived mineral substrate weathering and the release of nutrients into the proglacial environment as mineral‐bearing debris melts out from the ice (Cook et al., 2011; Rime et al., 2016). Subglacial environments are characterized by low temperature, absence of light, oligotrophic conditions, and high mineral content (Bakermans & Skidmore, 2011; Montross et al., 2014; Yde et al., 2010), which suggests that chemolithotrophic metabolisms could play a fundamental role in subglacial ecosystems (Boyd et al., 2014; Christner et al., 2014; Mitchell et al., 2013).

The focus of this work was on microbial diversity of subglacially derived basal ice (BI) and its broader significance within glacial and deglaciating systems. BI inherits physical and chemical characteristics from its close interaction with the glacier substrate, which differ from those of atmospherically derived (i.e., firnified) englacial ice (Hubbard et al., 2009; Swift et al., 2018). Specifically, BI is commonly characterized by high sediment content, enrichment in certain ions, and low bubble content (Hubbard et al., 2009; Knight, 1997). Differences in BI physical and chemical characteristics are often interpreted to indicate formation by distinct subglacial processes (Cook et al., 2007; Hubbard & Sharp, 1995; Knight, 1997; Knight & Knight, 1994; Swift et al., 2018).

**FIGURE 1:**
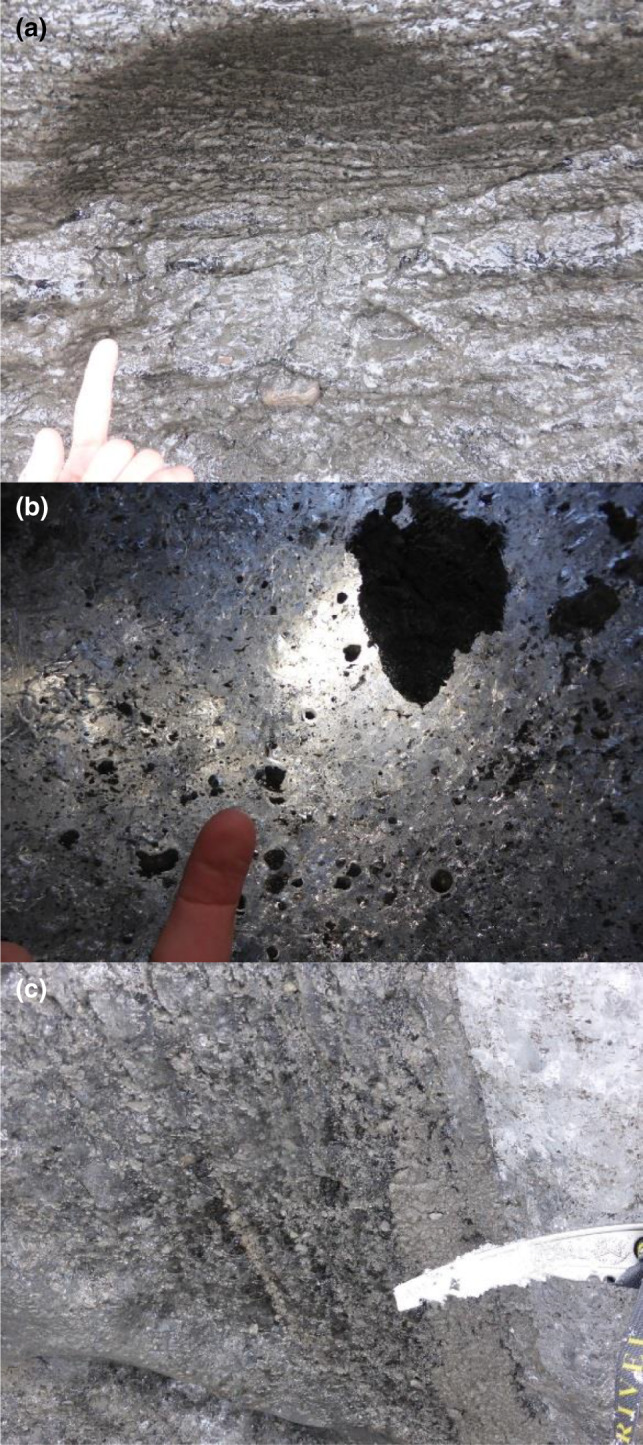
Examples of basal ice (BI) facies collected from Svínafellsjökull: a) stratified facies (S), rich in fine (clay/silt) sediment, with sediment arranged in angular aggregates; at a centimeter to decimetre scale, stratified facies appears layered, as the name suggests; b), dispersed facies (D) comprising dispersed aggregates of polymodal sediment; c), debris band (B), composed of sub‐vertically layered alternations between clear, bubble‐free ice and polymodal sediment—note also the white and bubble‐rich englacial ice to the right.

**FIGURE 2:**
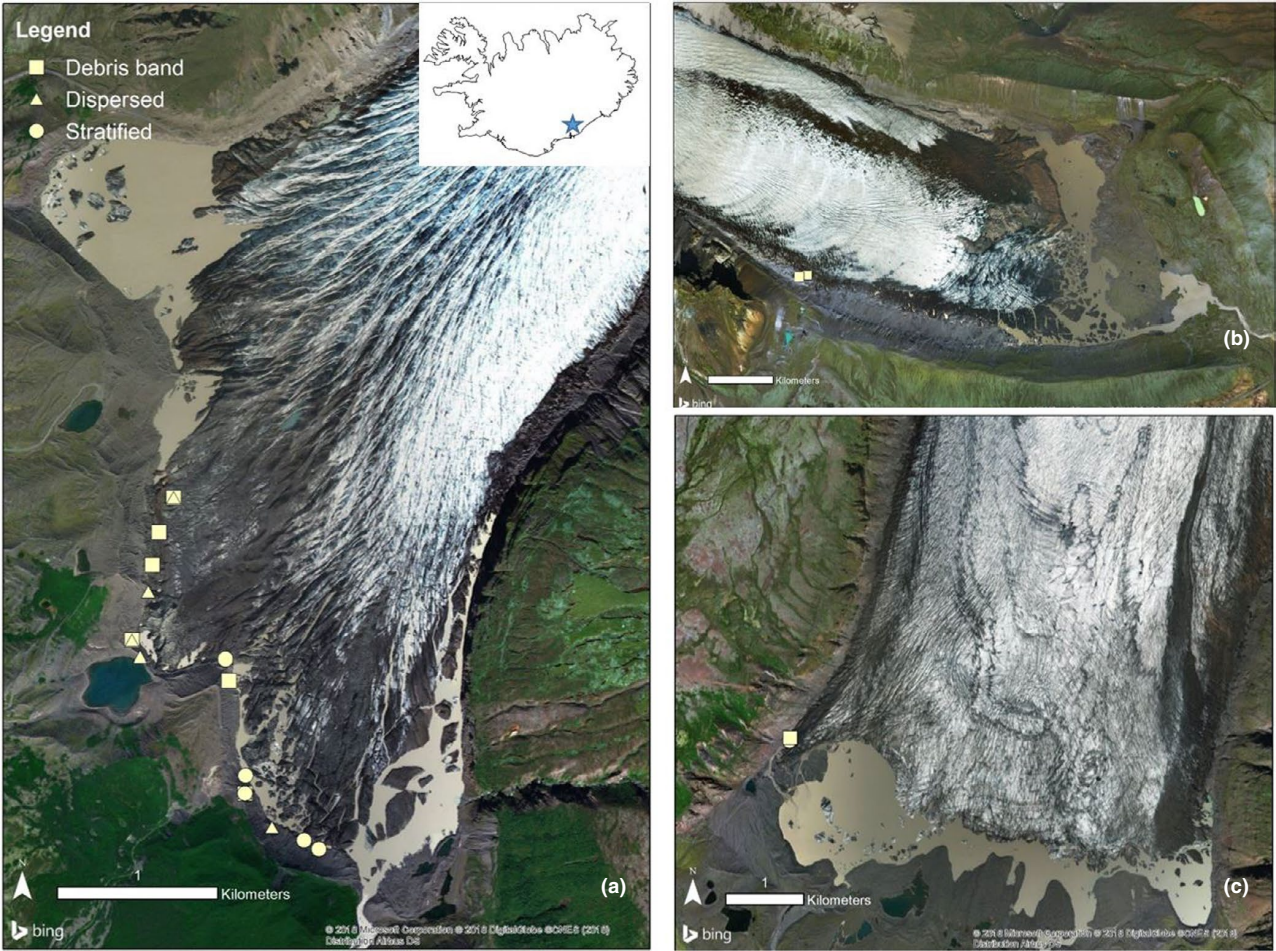
Map of the sampling points. Three glaciers were analyzed in this study: Svínafellsjökull (A), Kvíárjökull (B), and Skaftafellsjökull (C). Different shapes indicate different ice facies sampled.

BI is commonly split into different facies based on distinct physical and chemical characteristics (Hubbard et al., 2009; Knight, 1997). Three such facies are relevant to this study: stratified, dispersed, and debris bands. Stratified facies (Figure 1a) is stratigraphically the lowermost layer of basal ice and is found mainly in the southern part of Svínafellsjökull, in both western and eastern parts of Skaftafellsjökull, and the southern part of Kvíárjökull (locations shown in Figure 2), but is absent around much of the glacier margins (Cook et al., 2010; Ebert, 2003). It is characterized by its layered appearance and high debris content (5%–80% by volume) (Cook et al., 2007, 2010; Swift et al., 2018). Dispersed facies (Figure 1b) appears ubiquitously around glacier margins in sections of 3.5 m thickness on average and is characterized by low debris content (0.2%–2% by volume) and massive structure (Cook et al., 2011). Debris bands (Figure 1c) are characterized by discrete layers of debris‐laden stratified ice between bubble‐rich and debris‐poor firnified englacial ice or debris‐laden dispersed facies. Debris bands commonly exhibit high clast content. Depending on the nature of the subglacial environment and the sediment that exists there, clasts may exhibit evidence of glaciofluvial or subglacial wear, or both (i.e., sub‐angular to well‐rounded clasts with striations and/or facets) (Swift et al., 2018). The ice of different origins, composition, and provenance is hypothesized here to harbor distinct microbial communities as a result of natural selection and ecological processes. Knowledge of these communities in turn can contribute to the understanding of the evolution and biochemical functionality of glaciated landscapes.

Previous studies have demonstrated an abundance of microbial cells within BI. At Taylor Glacier, Antarctica, a BI cell content of 2.5 x 10^2^ to 1.2 x 10^4^ g ml^−1^ was found, and 7.9 x 10^6^ cells g^−1^ existed within basal sediment (Montross et al., 2014; Stibal et al., 2012); 8.7 x 10^5^ cells g^−1^ were found in Russell Glacier (Greenland) (Stibal, Hasan, et al., 2012); 1.7 – 6.8 x 10^5^ cells g^−1^ were found in Finsterwalderbreen, Svalbard (Lawson et al., 2015); and 1.3 – 1.4 x 10^7^ cells g^−1^ were found in Svínafellsjökull, Iceland (Toubes‐Rodrigo et al., 2016). Subglacial environments have been shown to foster an abundance of chemolithotrophic‐associated microorganisms (Boyd et al., 2014; Yde et al., 2010). For example, Mitchell et al., (2013) reported that bacterial Fe‐ and S‐ oxidizers were abundant in subglacial meltwater discharge from Robertson Glacier, Canada and that microorganisms were more abundant in minerals that could be easily oxidized, such as pyrite. Chemolithotrophic communities are not only important in subglacial environments (Boyd et al., 2014), but also play important roles in proglacial systems once released from the glacier (Frey et al., 2010). Methanogenic communities have been detected in subglacial environments, and the impact of the release of methane accumulated in these environments, including BI, can have a high impact on the global climatic system (Lawson et al., 2015; Stibal, Wadham, et al., 2012). The amount of organic carbon in freshly exposed proglacial sediments is a limiting factor for soil formation (Brankatschk et al., 2011). Until recently, it was thought that the main driver for the evolution from bare sediment into the soil was the deposition of microorganisms from atmospheric sources, such as wind, marine aerosols, or precipitation (Chuvochina et al., 2011; Temkiv et al., 2012; Womack et al., 2010). Nevertheless, recent research by Rime et al., (2016) indicated that BI has a substantial microbial input to the development of new soils. Our work builds upon these observations of the subglacial environment microbiota by characterizing the microbial communities and geochemical characteristics of the major ice types, enabling finer‐scaled interpretation of ecological interactions and biologically driven functionality within glaciers.

The potential microbial differences between BI facies and the relationship between geochemistry and microbial community in the BI are yet to be studied. To address these gaps, the primary aim of this study is to characterize the microbial diversity within distinct BI facies at three glaciers in southern Iceland. We hypothesize that distinctive ice facies, formed through different processes, will yield different microbial content and community compositions. The second aim of this study is to elucidate the potential microbiota–mineralogy relationship based on microbial diversity and geochemical analysis of the BI layer.

## 2 EXPERIMENTAL PROCEDURES

### 2.1 Study site and BI samples

The main focus of this study was Svínafellsjökull (n=16) (Figure 2a), and additional samples were also taken from nearby Kvíárjökull (n=2; Figure 2b) and Skaftafellsjökull (n=4; Figure 2c) to determine whether basal ice layers of different glaciers have similar or different microbiological properties. All three locations are temperate valley glaciers. On the first sampling campaign (April 2015), neither Skaftafellsjökull nor Kvíárjökull had accessible basal ice facies, but they were sampled during the second field campaign (May 2016). A sampling at each glacier targeted three distinct BI facies that have been identified in previous studies, namely debris band, dispersed, and stratified facies (Cook, Graham, et al., 2011; Cook et al., 2007, 2010; Cook, Swift, et al., 2011; Swift et al., 2006, 2018) (Figure 1). Accessible BI facies at Svínafellsjökull were sampled aseptically in April 2015 and May 2016 following procedures outlined by Toubes‐Rodrigo et al., (2016). The first 20–30 cm of BI was removed with an ice axe to prevent surface melt‐derived cross‐contamination. Sampled BI blocks were carved using flame‐sterilized chisels and carefully triple‐bagged in sterile plastic bags to prevent cross‐contamination from potential piercing (Toubes‐Rodrigo, Simon J Cook, et al., 2016). Individual BI samples were melted at ~4℃ in the bags (Stibal et al., 2015). Samples were allowed to settle, and soil and liquid fractions were separated by decanting. Samples for chemical analysis were oven‐dried at 60℃ overnight, while sediment samples for DNA extraction were processed immediately after decanting the liquid fraction.

### 2.2 Sediment chemical analysis

Total carbon (C), total nitrogen (N), total sulfur (S), and iron (Fe) were analyzed due to their relevance in chemolithotrophic metabolism (Madigan et al., 2010). No separation between organic and organic fractions was performed for this work. Sediment samples were vigorously shaken and mixed thoroughly to increase their homogeneity ahead of analysis. For total carbon and nitrogen, 0.2 grams of oven‐dried (60℃), BI sediment was subjected to dry combustion elemental analysis at 950˚C in a Leco TruSpec^TM^ instrument (Thermo Scientific, UK) (Macreadie et al., 2012). As a control, ethylenediaminetetraacetic in a solution containing 9.5 µg g^−1^of nitrogen and 41.1 µg g^−1^ of C was utilized. The precision of the techniques was 0.4% for C and 0.5% for N.

Fe and S analysis was performed using an iCAP 6000 series ICP‐OES (Thermo Scientific, UK) and microwave digestion. In short, 25 ml of an aqua regia was added to a preweighted sediment sample (0.5 g) and acid digested using a two‐step cycle at 90 and 170 ºC. Filtered samples were diluted to 50 ml and analyzed (Wavelengths and concentration ranges were as follows: Fe, 240.4 nm (Gomez et al., 2007) and 10–500 µg g^−1^; S, 180.7 nm (Santelli et al., 2008) and 0.5–50 µg g^−1^. Calibration standards were matrix‐matched, and method blanks were used to correct data. All results are expressed in part per million in mass (µg g^−1^). The precision of the technique was 2.0% for Fe and 2.5% for S.

### 2.3 Sediment single particle analysis (SPA)

Dried BI‐derived sediment (25 mg) aliquots were suspended in pure methanol and subjected to ultra‐sonication using an S‐Series Table Top ultrasonicator (Sonicor Inc. USA) at the highest power rating until a homogenous dispersion was observed. Fifty microlitre aliquots were transferred to Leit Adhesive Carbon Tabs 12 mm (Agar Scientific, UK) mounted on aluminum SEM Stubs 12 mm diameter, 6 mm pin (Agar Scientific, UK) in such a way that particles were separated from each other. Mounted stubs were subjected to computer‐controlled scanning electron microscopy coupled with energy dispersive X‐ray spectrometry (SEM‐EDX) using a Supra 40VP microscope controlled by SmartSEM software (Carl Zeiss Ltd, UK). For SEM, the acceleration voltage of 25kV was applied and samples viewed at a magnification of 2500X. For *in situ* elemental analysis, the SEM is equipped with a Backscattered Electron Detector (Apollo 40 SDD. EDAX Inc. USA) controlled by Genesis software (Laskin et al., 2006; Williams et al., 2013); optimization of the scanning time was performed, and the best results were obtained using a scanning time of 15 seconds per particle. The number of particles analyzed per sample ranged between 55 and 12489.

### 2.4 Raman analysis

Small amounts of sample were mounted on double‐sided carbon tape fixed onto glass microscope slides, removing the excess by tapping it on the side of the microscope slide. At least fifty particles per sample were analyzed using a Renishaw InVia Raman microscope fitted with a Peltier‐cooled charge‐coupled device detector. The source of excitement was a 514.5 nm Ar^+^ laser. The instrument was calibrated at the beginning of each set of analyses using a silicon chip. The instrument was used in extended mode obtaining spectral data ranging from 100 to 2200 wavenumbers. The number of acquisitions varied between 1 and 4‐ with 10‐second exposures to ensure an acceptable S/N ratio. The power density varied between 2 and 8 mW in the sample. Data acquisition was carried out with the WireTM and Spectracalc software packages from Renishaw. Spectral identification was done using an in‐house spectral library for the iron oxides, the RRUFF database, and a commercially available spectral library via Spectracalc software (GRAMS, Thermo Fischer, UK).

### 2.5 DNA extraction and sequencing

Total genomic DNA was extracted using MoBio kits. For the 2015 samples, the PowerSoil® kit was used to extract DNA on 0.25g (wet weight) samples of BI sediment. However, as relatively low DNA yields were achieved, the PowerMax® Soil DNA Isolation kit was employed in the 2016 campaign allowing DNA extraction from up to 10 g of wet sediment (Elser et al., 2015; Fernández‐Martínez et al., 2016). DNA extracts were maintained at −20℃ before analysis. Samples were amplified for sequencing in a two‐step process. The forward primer was constructed with the Illumina i5 sequencing primer (5’‐TCGTCGGCAGCGTCAGATGTGTATAAGAGACAG‐3’) and the 357F primer (5’‐CCTACGGGNGGCWGCAG‐3’). The reverse primer was constructed with the Illumina i7 sequencing primer (5‐GTCTCGTGGGCTCGGAGATGTGTATAAGAGACAG‐3’) and the 785R primer (5’‐GACTACHVGGGTATCTAATCC‐3’) (e.g., (Diao et al., 2017)). Amplifications were performed in 25 μl reactions with the Qiagen HotStar Taq master mix (Qiagen Inc, US), 1 μl of each 5uM primer, and 1 μl of the template. Reactions were performed on ABI Veriti thermocyclers (Applied Biosystems, US) under the following thermal profile: 95˚C for 5 min, then 10 cycles of 94˚C for 30 secs, 50˚C for 40 secs (+0.5˚C per cycle), 72˚C for 1 min, followed by 25 cycles of 94˚C for 30 secs, 54˚C for 40 secs, 72˚C for 1 min, and finally, one cycle of 72˚C for 10 min and 4˚C hold.

Amplification products were visualized with eGels (Life Technologies, Grand Island, New York). Products were then pooled equimolar, and each pool was size selected in two rounds using Agencourt AMPure XP (BeckmanCoulter, Indianapolis, Indiana) in a 0.75 ratio for both rounds. Size selected pools were then quantified using the Qubit 2.0 fluorometer (Life Technologies, Thermo Fisher, UK) and loaded on an Illumina MiSeq (Illumina, US) 2x300 flow cell at 10 pM. The average number of reads per sample was 10K.

### 2.6 Sequence classification

Raw sequence reads (FastQ) were trimmed, aligned, and filtered using Mothur (Kozich et al., 2013). Sequences that diverged from the sequence length median were removed. Chimeras were removed using uchime included in Mothur. Sequences were aligned and processed using Parallel‐META 3.4.1 pipeline (Jing et al., 2017) to analyze the paired‐end 16S rRNA ​gene sequences. Phylum and Genera were the taxonomic levels analyzed and reported. For the operational taxonomic unit (OTU) clustering, a threshold of 97% homology was chosen (Jing et al., 2017). Taxonomic identification of OTUs was performed using the SILVA database built‐in Parallel‐META 3.4.1 (v123). The Phyla, Genera, and OTU tables generated were used for further analysis. The minimum sequence count threshold was 2; abundance thresholds were fixed for maximum 0.1% and minimum 0%, minimum no‐zero abundance threshold 10%, and minimum average abundance threshold 0.1% (Jing et al., 2017).

### 2.7 Functional prediction from community data

The PICRUSt v1.1.13 pipeline was used to predict the functional metabolic potential of the communities (Langille et al., 2013) inhabiting BI. OTUs were classified against the Green Genes database (version 13_5) (DeSantis et al., 2006). Copy number was normalized, and the metagenome functional profiles were predicted, generating a table of Kyoto Encyclopedia of Genes and Genomes (KEGG) Orthologs (KOs). The resulting table was collapsed at KO level 1, to give a broad assessment of the general functionality of the metagenome, and level 3, filtering for metabolism (Wang et al., 2016).

### 2.8 Statistical analysis

All data analysis was performed using R (version 4.0.2) combined with ggplot2 (Wickham, 2009) and igraph (Csardi & Csardi, 2007) packages for graphical visualization. Samples were assumed to not be normally distributed, and the Kruskal–Wallis test was performed using the pgrimess package (Giraudoux, 2018). Samples were considered to differ significantly when *p*‐value <0.05.

To visualize potential relationships between bacteria genera, a correlation matrix was produced. To calculate the correlation coefficients, the package Hmisc (Harrell Jr, 2013) was used to generate a matrix that was imported into the package igraph (Csardi & Csardi, 2007). Network visualization of highly correlated taxa (*r* > 0.5) (Csardi & Csardi, 2007; Csardi & Nepusz, 2006) was produced. The length of the edges connecting the nodes of the plot is inversely proportional to the r‐coefficient, and the size of the nodes is logarithmically proportional to the abundance of the taxon. Different colors were given to the different genera based on their potential metabolisms based on a literature search.

## 3 RESULTS

### 3.1 Geochemistry of BI sediment

**FIGURE 3:**
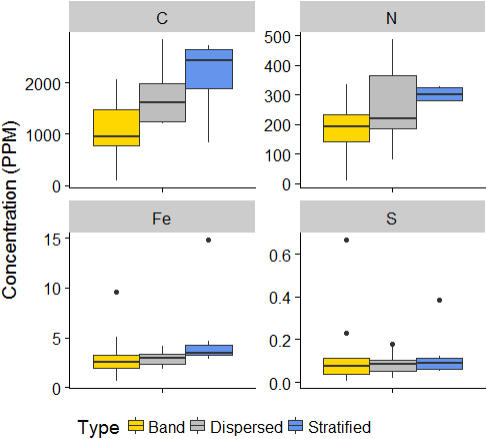
Box plot showing the concentrations (in µg g^−1^) of the different elements analyzed from sediment entrained in basal ice (BI). C and N were analyzed by Leco TruSpec, whereas Fe and S were analyzed by ICP‐OES. Lines represent the median and the upper and lower limit of the box represent the 75 and 25 percentiles, respectively. Dots represent the outliers.

Figure 3 illustrates total C, N, S, and Fe in the sediment in basal in debris bands dispersed and stratified facies. No statistically significant differences (p‐value>0.05) were found between the concentrations of elements in sediment entrained within different BI types.

Total C in BI sediment was consistently low across all facies but ranged from 82 µg g^−1^ in a debris band sample (B6‐16) to 2833 µg g^−1^ in a dispersed facies sample (D3‐16). On average, C concentrations were lowest in debris bands (1046.5 ± 668.6 µg g^−1^; n=5), followed by dispersed facies (1748.0 ± 637.2 µg g‐1; n=6), and stratified facies (2102.3 ± 874.0 µg g^−1^; n=4).

Total N concentrations in BI sediment were very low in all samples and ranged from 8 µg g^−1^ in debris band sample B1‐16 to 486 µg g^−1^in dispersed facies sample D3‐16. On average, N concentrations were lowest in debris bands (189.5 ± 109.3 µg g^−1^), followed by dispersed facies (265.5 ± 152.2 µg g^−1^), and stratified facies (302.8 ± 26.9 µg g^−1^). Fe concentration ranged from 0.6 µg g^−1^ in debris band sample B6‐16 to 14.8 µg g^−1^in stratified facies sample S1–15. On average, Fe values were lowest in dispersed facies (2.9 ± 0.8 µg g^−1^), followed by debris bands (3.3 ± 2.6 µg g^−1^), and stratified facies (5.1 ± 4.3 µg g^−1^). Total S values were very low, ranging from 4.8 x 10^−3^ µg g^−1^in debris band sample B6‐16 to 0.7 µg g^−1^in debris band sample B4‐15. On average, S concentration values were lowest in dispersed facies (0.09 ± 0.05 µg g^−1^), followed by stratified facies (0.13 ± 0.12 µg g^−1^), and debris bands (0.14 ± 0.20 µg g^−1^).

**FIGURE 4:**
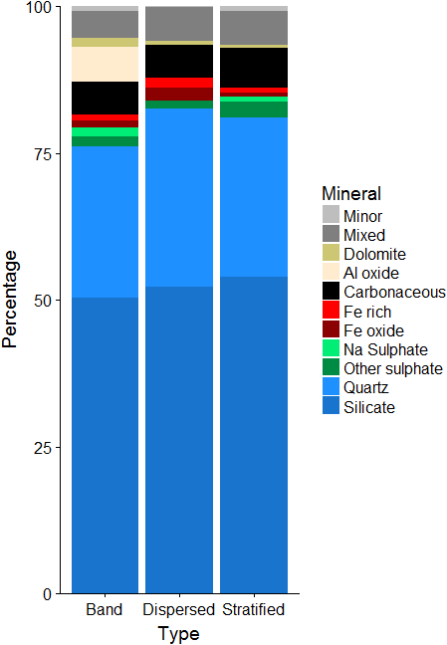
Mineralogical analysis of the sediment entrapped in basal ice (BI) using automated Single Particle Analysis (SPA) by Scanning Electron Microscopy coupled with Energy Dispersive X‐ray (SEM‐EDX). Particles were classified using a combination of the cluster chemical boundaries defined by Kandler et al., (2011) and (Anaf et al., 2012).

The glaciers in this study drain the Öræfajökull ice cap, which covers a stratovolcano. The bedrock is dominated by basaltic lavas, olivine porphyritic lavas, with minor components of hyaloclastite, and subglacially erupted cube jointed basic to intermediate volcanic rock with hyaloclastic layers (Helgason & Duncan, 2001). The SPA, performed following the chemical boundaries defined in Kandler et al. (Kandler et al., 2007) (Figure 4), showed sediment mineralogy dominated by silicates (50.2% in debris bands, 52.1% in dispersed, and 53.8% in stratified). Carbonaceous particles—which encompass all C‐rich particles—appeared consistently among the analyzed samples (5.6% in debris bands, 5.6% in dispersed, and 6.7% in stratified). Fe‐rich and Fe‐oxides particles were abundant in the SPA (2.2% in debris bands, 3.9% in dispersed, and 1.5% in stratified). Although not as abundant, sulfate particles also appeared among the samples (3.3% in debris band, 1.4% in dispersed, and 3.6% in stratified). No statistically significant differences were observed between samples (p‐value >0.05). Mixed particles, which represent those particles that could not be resolved to specific mineralogy, represented a significant proportion of the analyzed particles (4.7–5.9% of the total).

Silicates were dominated by olivine, feldspars (e.g., sanadine, oligoclase, and anorthosite), and quartz. Fe‐rich particles analyzed by micro‐Raman corresponded to martite, hematite, magnetite, and pyrrhotite. Carbonates identified by micro‐Raman were calcite, aragonite, and rhodochrosite. Some of the minor minerals corresponded to apatite, titanite, and libethenite.

### 3.2 Bacterial diversity of BI sediment

**FIGURE 5:**
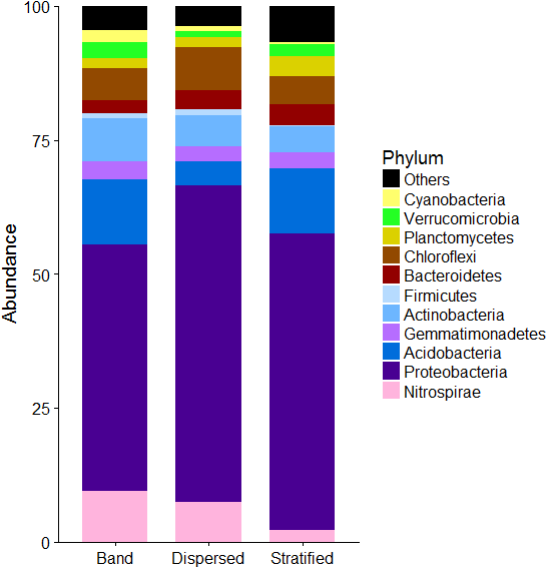
Relative abundance of 16S rRNA gene sequences classified to phylum level in the three basal ice (BI) types over the 2015 and 2016 sampling campaigns at Svínafellsjökull. Based on 16S rRNA gene sequence‐derived OTU table analysis in Parallel Meta 3.4.1. Phyla below 1% abundance were reported with genera below this threshold and designated as a single group termed “Others.”

**FIGURE 6:**
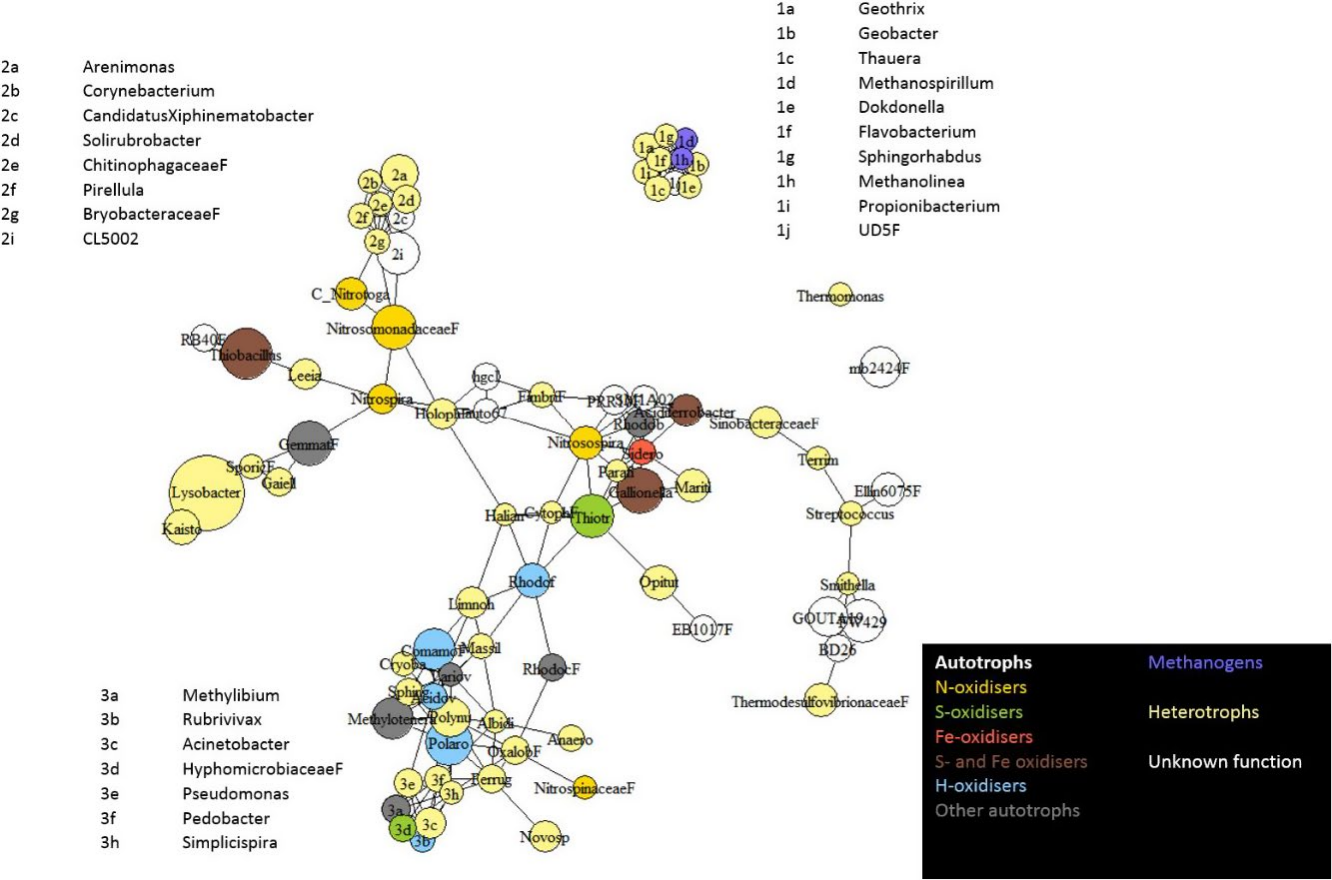
Bacterial (16S rRNA gene) network analysis and inferred metabolic groupings associated with sediment entrained within BI facies. The size of the circles is proportional to the mean abundance of the bacterial taxonomic group. Color‐coding highlights inferred functional categories based on the literature.

BI facies‐specific bacterial 16S rRNA gene‐based phyla abundance is presented in Figure 5. The most abundant phyla were Proteobacteria (debris bands 46.6%, dispersed 62.9%, and stratified 58.0%), Acidobacteria (debris bands 11.0%, dispersed 3.5%, and stratified 10.6%), Chloroflexi (debris band 6.2%, dispersed 7.6%, and stratified 5.2%), Actinobacteria (debris band 5.7%, dispersed 5.0%, and stratified 4.7%), and Nitrospirae (debris bands 6.9%, dispersed 6.2%, and stratified 1.6%). Acidobacteria showed significant differences between ice types (Kruskal–Wallis p‐value <0.05), being significantly less abundant in dispersed facies than in stratified facies or debris bands, although no differences were observed between debris bands and stratified ice (Figure 6).

Table 1 shows genera of an abundance over 1%. A Tukey test revealed that the only statistically significant difference observed was for stratified facies, where *Thiobacillus* was more abundant (7.5 ± 3.7%) than in dispersed facies.

**TABLE 1:** Basal ice specific relative mean percentage abundance ±standard deviation of the major bacterial genera (>1% total abundance). *p*‐values for different tests were calculated based on sample distribution. When samples were normally distributed, ANOVA was used to analyze for statistically significant differences; otherwise, Kruskal–Wallis was utilized

	Debris band	Dispersed	Stratified	Kruskal–Wallis *p*‐value
*Lysobacter*	9.7 ± 7.1	13.0 ± 10.3	12.1 ± 10.8	0.808
*Thiobacillus*	3.0 ± 3.0	3.3 ± 3.3	7.5 ± 3.7	0.046*
*Polaromonas*	1.2 ± 1.4	6.2 ± 7.2	0.6 ± 0.6	0.236
*Gallionella*	1.6 ± 1.5	2.6 ± 2.2	5.5 ± 3.7	0.088
*Methylotenera*	0.8 ± 1.1	4.0 ± 4.3	1.1 ± 0.6	0.178
*Polynucleobacter*	1.0 ± 2.4	3.4 ± 4.8	0.1 ± 0.1	0.598
*Arenimonas*	2.8 ± 2.3	1.0 ± 1.6	1.0 ± 1.1	0.066
*Kaistobacter*	1.1 ± 1.0	1.3 ± 1.2	1.1 ± 1.3	0.981
*Rhodoferax*	1.4 ± 1.8	1.0 ± 0.7	1.0 ± 0.6	0.931

* Significant (*p*‐value <0.05).

### 3.3 Network analysis

**FIGURE 7:**
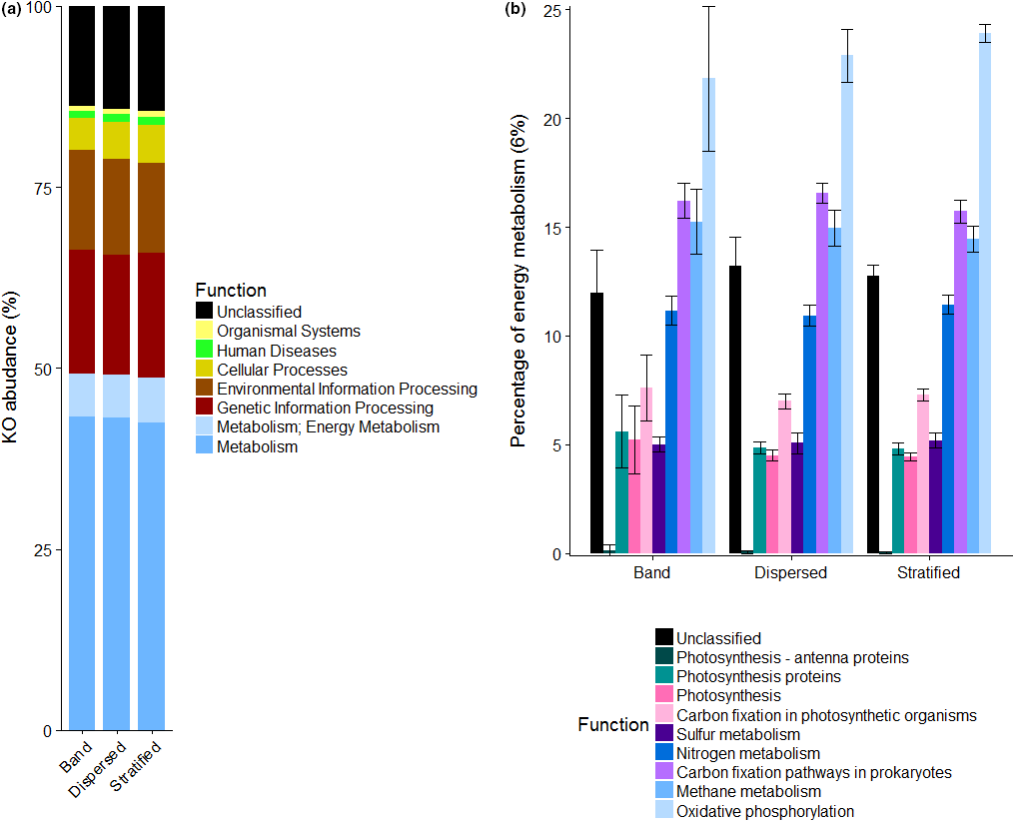
Potential functionality predicted by PICRUSt. A) General functions, B) Close‐up to potential energy metabolic pathways, representing ~6% of the total of the KOs predicted.

Network analysis showed two main groups: one containing the majority of taxa and a smaller group that contained only minor groups (Figure 7). Two taxa showed no significant relationships (r‐coefficient >0.5): the family mb242 and *Thermomonas*. None of the chemolithotrophic‐affiliated taxa occupied terminal positions in the network analysis, and in general, they were linked to heterotrophic genera (e.g., *Thiobacillus*—*Leeia*, *Gallionella*—*Maritimonas*).

Most iron oxidizers (*Gallionella*, *Acidiferrobacter*, and *Sideroxidans*) appeared clustered together in the main cluster, showing positive correlations. These genera co‐occurred with other chemolithotrophic‐containing genera (*Thiobacillus*—S oxidizer; *Ntrosospira*—N oxidizer, *Rhodobacter*—H oxidizers, among other metabolisms). OTUs belonging to genera containing hydrogen oxidizers (*Comamonas*, *Rhodoferax*, and *Acidovorax*) co‐occurred with other potential autotrophs with a variety of autotrophic metabolisms (*Polaromonas* and *Variovorax*—genera known to have versatile metabolisms, including hydrogen oxidation, *Methylotenera*—methylotroph), as well as heterotrophs (*Sphingomonas*, *Massilia*, and *Cryobacter*).

Two OTUs affiliated with genera containing hydrogenotrophic methanogenic archaea (*Methanolinea* and *Methanospirilum*) were detected in a second minor cluster, co‐occurring with potential heterotrophs, such as *Geothrix*, *Geobacter*, *Thauera*, and *Dokdonella*.

### 3.4 Potential metabolic pathways in glacier BI

The PICRUSt KEGG Orthologue (KO) analysis identified a high abundance of diverse metabolism‐related metagenomes in BI, together with genetic information and environmental information processing. The most abundant functional groups predicted were “Metabolism” representing ~50% in all cases, followed by “Genetic Information Processing” and “Environmental information processing.” Due to the oligotrophic nature of the BI environment, an analysis of predicted pathways for obtaining energy was undertaken. The most abundant pathways were oxidative phosphorylation (>20% on average for the BI types), carbon fixation (>15%), and methane metabolism (~15%).

Slightly less abundant, but still at relevant levels, nitrogen metabolism pathways are represented by ~12% of the total predicted pathways. Photosynthesis‐related pathways represent a minor part of the predicted results, being photosynthesis itself only ~5%, and assisting pathways, such as antenna protein, <1%), photosynthesis proteins, ~5%. Whereas non‐photosynthetic C fixation predicted pathways represented over 15% of the total predicted metabolisms consistently on all BI types, C fixation pathways in photosynthetic organisms only represented ~7.5%. Around 5% of the total predicted pathways corresponded to sulfur metabolism.

## 4 DISCUSSION

This paper aimed to elucidate two crucial geomicrobiological properties of BI in our target temperate Icelandic glaciers, namely (i) to characterize the BI bacteriome to identify whether there are significant differences among physically distinctive ice facies and (ii) knowing that BI is an active ecosystem (Kayani et al., 2018), to propose the likely biogeochemical pathways that fuel that ecosystem.

### 4.1 Microbial differences among BI facies

Previous research on BI has classified ice facies based on physicochemical characteristics to ascertain their respective origins under the premise that different characteristics are likely to represent different origins (e.g., (Cook et al., 2010; Cook, Swift, et al., 2011; Hubbard et al., 2009; Hubbard & Sharp, 1995; Knight, 1997; Swift et al., 2018)). Such work, typically undertaken at the ice margin, provides important information about the otherwise inaccessible subglacial environment, crucial to our understanding of glacier ecology. We predicted that different BI facies would host different microbial communities and abundances because of their different physicochemical properties.

One phylum (Acidobacteria) and one bacterial genus (*Thiobacillus*) showed significant differences between facies. Acidobacteria were more abundant in both stratified facies and debris bands than in dispersed facies, and *Thiobacillus* was more abundant in stratified facies than dispersed facies, but not more abundant than in the debris bands (Table 1). These results show that there are microbiological differences between ice types, especially between stratified facies and dispersed facies, that can be linked to their different origins, agreeing with observations by Swift et al., (2018). It is generally acknowledged that stratified facies form by the freeze‐on of subglacial meltwaters beneath the glacier terminus and, specifically in the case of Icelandic glaciers, through glaciohydraulic supercooling (Cook et al., 2007, 2010; Hubbard et al., 2009). Dispersed facies, on the other hand, is thought to be derived from further up‐glacier through tectonic and strain‐induced metamorphism of the lower parts of ogive (debris) bands (Cook, Swift, et al., 2011). Our data lend some support to these hypotheses that stratified and dispersed facies are formed through very different processes. Several studies have hypothesized that debris bands are related in some way to stratified ice based on their similar physical and chemical characteristics (Cook et al., 2010; Hubbard et al., 2009; Swift et al., 2018), and again, our data lend support to that idea.

### 4.2 Iron and hydrogen fuel the BI ecosystem

Total carbon concentration in the sediment was very low (debris band: 1046 µg g^−1^, dispersed: 1748 µg g^−1^, and stratified: 2102 µg g^−1^) (Figure 3) compared to soils of other liquid water‐limited ecosystems (e.g., hyperarid deserts (Valdivia‐Silva et al., 2012)). Total C concentration in the recently deglaciated soils of the surrounding area has been previously reported to be on the same order of magnitude as our results (~500 µg g^−1^) (Vilmundardóttir et al., 2015).

Two possibilities for the presence of total C in basal ice are feasible: Ives (2007) illustrated how the terminus of Svínafellsjökull was ~2 km further up‐valley between the 14th and 19th centuries. The former glacier foreland, now covered by ice, was used for agricultural purposes, which implies that soil must have been rich in C. It was also reported that the ice margin of Skaftafellsjökull was mined for birchwood by early settlers in Iceland. In both instances, the glacier termini advanced over these carbon‐rich landscapes to reach their Little Ice Age maximum extents in the 19th Century (Helgason & Duncan, 2001). These carbon‐rich agricultural soils and woodland would have become entrained into the BI layer (Cook et al., 2007, 2010). The same process was used to explain the high cell counts in sediment entrained within BI observed by Toubes‐Rodrigo et al. (2016). The other possibility is that C‐fixation via chemolithotrophy occurs over time, leading to an increase in C linked to the oxidation of minerals. Oxidized minerals were detected by both Raman (Fe‐oxides, such as pyrrhotite or magnetite) and SPA (Fe‐oxides and sulfates).

Acknowledging that PICRUSt metabolic modeling is not exempt from caveats and that this simulation is not a substitute for metatranscriptomics, we decided to use it to build a hypothesis of the potential metabolism present in BI. Figure 7 shows that the predicted metabolism in BI is dominated by chemosynthesis over photosynthesis (16.2% vs 7.3% of energy‐related KOs; Welch *t‐*test *p*‐value <0.01), which is not surprising given the lack of light in the subglacial environment (Yde et al., 2010). Recent research has shown a high abundance of genes associated with ribulose bisphosphate carboxylase (RuBiSCO) in BI, which supports our hypothesis that BI is driven by chemolithotrophy (Kayani et al., 2018). Based on the abundance of Fe‐rich particles and silicates, and the abundance of microorganisms with Fe‐oxidizing capabilities (*Thiobacillus*, *Gallionella*) and H‐oxidizing capabilities (*Polaromonas*, *Rhodoferax*), we suggest that the BI ecosystem is sustained by the oxidation of Fe‐particles, and oxidation of silicate comminution‐derived H_2_ (Telling et al., 2015), especially when reduced Fe is depleted. Additionally, it has been suggested that iron is basalts can be linked to the production of H_2_ in subsurface ecosystems (Stevens & McKinley, 2000). Members of genera Thiobacillus and Rhodoferax have been isolated from hydrogenotrophic enrichment cultures from basaltic bedrock Icelandic glaciers, which adds extra support to our hypothesis (Dunham et al., 2021). In Durham et al., (2021), subglacial sediment showed higher H_2_ oxidation levels in inoculated samples, indicating that H_2_ would play an important role in subglacial microbial ecology. In Kötlujökull glacier—basaltic bedrock, as the glaciers in this study—hydrogen was suggested to be the main reductant for chemolithotrophy (Dunham et al., 2021). Members of the genus *Rhodoferax* have been isolated from hydrogenotrophic enrichment cultures from Kötlujökull (Dunham et al., 2021). Analysis of the microbiome of other glaciers, such as the Matanuska glacier, also showed a high abundance of members of presumably active families (analyzed by RNA/DNA ratio) that have been identified in this work, namely: *Comamonadaceae* (which includes *Polaromonas* and *Rhodoferax*), *Gallionellaceae* (which includes *Gallionella*), and *Methylophilaceae* (including *Methylotenera*) (Kayani et al., 2018). However, until metatranscriptomics/metagenomic data from BI have been retrieved and analyzed, this conclusion remains a hypothesis.

Whereas Fe‐ and S‐ oxidation have been previously reported as potential metabolism supporting subglacial environments, results from this work indicate that H‐oxidation could be of vital importance in subglacial environments, as suggested by Telling et al., (2015). H_2_ oxidation is known to support life in subsurface ecosystems (Bagnoud et al., 2016; Chapelle et al., 2002) and has been proposed as the main reaction to support life in extraterrestrial planets and moons, such as Mars (McMahon et al., 2016) or Enceladus (Seewald, 2017).

Several clusters of chemolithotrophic and heterotrophic bacteria were identified in the network analysis (Figure 7), suggesting that BI is a cooperative environment in which C fixed by autotrophic bacteria can then be utilized by heterotrophic organisms (e.g., *Lysobacter*, *Kaistobacter*). Additionally, heterotrophic bacteria and especially anaerobic respiration can help to replenish substrates for autotrophs (Macey et al., 2020). A very tightly clustered group, comprising bacteria and archaea, albeit at low abundance, was identified in the network analysis (Figure 7). Although archaea were not the main focus of this study, 16S rRNA genes affiliated with two archaeal methanogens—*Methanolinea* and *Methanospirillum*—were recovered. These two genera co‐occurred in the same cluster with bacteria presenting different metabolisms: aerobic (*Dokdonella*, *Flavobacterium*, *Sphingorhabdus*), facultative anaerobic (*Thauera*), anaerobic (*Geobacter*), and fermentative (*Geothrix*) (Coates et al., 1999). The presence of fermenting bacteria can generate substrates used by methanogens, such as acetic acid for acetoclastic methanogenesis (Stibal, Wadham, et al., 2012, Wadham et al., 2012). On the other hand, the presence of H_2_ from fermentative processes and silicate comminution can be utilized by hydrogenotrophic methanogens as a substrate (Telling et al., 2015). In addition, methane metabolism was predicted among the most abundant metabolic pathways from PICRUSt data, which not only related to methanogenesis but also methanotrophy. A high abundance of methanotrophic bacteria was identified in BI, such as *Methylotenera* (Table 1) (Mustakhimov et al., 2013). The presence of tightly associated clusters of anaerobes and aerobes could be indicative of the presence of biofilms whereby aerobic species form the outermost part of the biofilm where they consume oxygen, which in turn allows the proliferation of anaerobic organisms in the inner part of the biofilm; this also results in mineralogical heterogeneity of the biofilm (Brown et al., 1994).

## 5 CONCLUSIONS

BI from southern Icelandic glaciers contains a diverse community of bacteria. Different ice facies were analyzed separately and results indicate that BI types support distinctively different bacterial communities: stratified and dispersed facies being two end members, and debris bands occupying an intermediate position. This is consistent with similar differences found based on the chemical and physical composition of the BI (Swift et al., 2018). Toubes‐Rodrigo et al., (2016) estimated that 10^16^ cell yr^−1^ were released from the BI at Svínafellsjökull, and demonstrated the capacity of some of these microorganisms to proliferate at low temperatures. To sustain such a high cell number, we hypothesize that BI sediment is enriched in C as a consequence of subglacial chemolithotrophy, strongly linked to the mineralogy of the environment as previous research has shown (Boyd et al., 2014; Kayani et al., 2018; Mitchell et al., 2013). Dispersed facies showed the highest abundance of silicates compared to debris bands and stratified facies. Silicates can produce H_2_ in subglacial ecosystems (Telling et al., 2015), which could support a microbial ecosystem based on hydrogenotrophy, and the abundance of H‐oxidizing *(Polaromonas*) bacteria was greatest in dispersed facies, suggesting that silicate comminution could act as fuel for microorganisms, upon depletion of other reduced substrates, such as Fe (II), that are more abundant in stratified and debris bands, and could be used by Fe‐oxidisers, such as *Thiobacillus*. Network analysis revealed a tight co‐occurrence of these C‐fixing bacteria with heterotrophs (e.g., *Lysobacter*, *Kaistobacter*), which can feed on the C fixed by mineral oxidizers. The presence of an active mineral‐oxidizing microbiota is likely to increase the weathering rates in BI, releasing nutrients, but in the process increasing the fixed organic C content due to chemolithotrophy. The melt‐out of BI at the ice margin will release carbon, other nutrients, and active microbiota to the ice‐marginal environment, thereby promoting soil formation as glaciers undergo recession (Rime et al., 2016).

## 6 ETHICS STATEMENT

Fieldwork in Iceland was conducted under permit from The Icelandic Centre for Research, Rannis (Research Declaration Nr. 1/2016).

## CONFLICT OF INTEREST

None declared.

## AUTHOR CONTRIBUTIONS

Mario Toubes‐Rodrigo involved in conceptualization, formal analysis, methodology, visualization, and writing–original draft. Sanja Potgieter‐Vermaak involved in formal analysis, investigation, methodology, writing–review and editing. Robin Sen involved in conceptualization, funding acquisition, supervision, writing–review and editing. Edda Oddsdóttir involved in resources. David Elliott involved in funding acquisition, supervision, and writing–review and editing. Simon Cook involved in conceptualization, funding acquisition, project administration, supervision, and writing–review and editing.

## Data Availability

The raw sequencing data are available in the NCBI database under the BioProject accession number PRJNA678168: https://www.ncbi.nlm.nih.gov/bioproject/PRJNA678168

## References

[mbo31200-bib-0001] Anaf, W. , Horemans, B. , Van Grieken, R. , & De Wael, K. (2012). Chemical boundary conditions for the classification of aerosol particles using computer controlled electron probe microanalysis. Talanta, 101, 420–427. 10.1016/j.talanta.2012.09.051 23158343

[mbo31200-bib-0002] Anesio, A. M. , Hodson, A. J. , Fritz, A. , Psenner, R. , & Sattler, B. (2009). High microbial activity on glaciers: Importance to the global carbon cycle. Global Change Biology, 15, 955–960. 10.1111/j.1365-2486.2008.01758.x

[mbo31200-bib-0003] Bagnoud, A. , Chourey, K. , Hettich, R. L. , de Bruijn, I. , Andersson, A. F. , Leupin, O. X. , Schwyn, B. , & Bernier‐Latmani, R. (2016). Reconstructing a hydrogen‐driven microbial metabolic network in Opalinus Clay rock. Nature Communications, 7, 12770. 10.1038/ncomms12770 PMC5067608 27739431

[mbo31200-bib-0004] Bakermans, C. , & Skidmore, M. L. (2011). Microbial metabolism in ice and brine at ‐5??C. Environmental Microbiology, 13, 2269–2278. 10.1111/j.1462-2920.2011.02485.x 21535342

[mbo31200-bib-0005] Boyd, E. S. , Hamilton, T. L. , Havig, J. R. , Skidmore, M. L. , & Shock, E. L. (2014). Chemolithotrophic primary production in a subglacial ecosystem. Applied and Environmental Microbiology, 80, 6146–6153. 10.1128/AEM.01956-14 25085483 PMC4178699

[mbo31200-bib-0006] Bradley, J. A. , Arndt, S. , Sabacká, M. , Benning, L. G. , Barker, G. L. , Blacker, J. J. (2016). Microbial dynamics in a High Arctic glacier forefield: A combined field, laboratory, and modelling approach. Biogeosciences, 13, 5677.

[mbo31200-bib-0007] Brankatschk, R. , Töwe, S. , Kleineidam, K. , Schloter, M. , & Zeyer, J. (2011). Abundances and potential activities of nitrogen cycling microbial communities along a chronosequence of a glacier forefield. The ISME Journal, 5, 1025. 10.1038/ismej.2010.184 21124490 PMC3131848

[mbo31200-bib-0008] Brown, D. A. , Kamineni, D. C. , Sawicki, J. A. , & Beveridge, T. J. (1994). Minerals associated with biofilms occurring on exposed rock in a granitic underground research laboratory. Applied and Environmental Microbiology, 60, 3182–3191. 10.1128/AEM.60.9.3182-3191.1994 16349374 PMC201787

[mbo31200-bib-0009] Chapelle, F. H. , O'Neill, K. , Bradley, P. M. , Methé, B. A. , Ciufo, S. A. , Knobel, L. R. L. , & Lovley, D. R. (2002). A hydrogen‐based subsurface microbial community dominated by methanogens. Nature, 415, 312–315. 10.1038/415312a. 11797006

[mbo31200-bib-0010] Christner, B. C. , Priscu, J. C. , Achberger, A. M. , Barbante, C. , Carter, S. P. , Christianson, K. , … Vick‐Majors, T. J. (2014). A microbial ecosystem beneath the West Antarctic ice sheet. Nature, 512, 310–313. 10.1038/nature13667 25143114

[mbo31200-bib-0011] Chuvochina, M. S. , Marie, D. , Chevaillier, S. , Petit, J.‐R. , Normand, P. , Alekhina, I. A. , & Bulat, S. A. (2011). Community variability of bacteria in alpine snow (Mont Blanc) containing Saharan dust deposition and their snow colonisation potential. Microbes and Environments, 26, 237–247. 10.1264/jsme2.ME11116 21666389

[mbo31200-bib-0012] Coates, J. D. , Ellis, D. J. , Gaw, C. V. , & Lovley, D. R. (1999). Geothrix fermentans gen. nov., sp. nov., a novel Fe (III)‐reducing bacterium from a hydrocarbon‐contaminated aquifer. International Journal of Systematic and Evolutionary Microbiology, 49, 1615–1622. 10.1099/00207713-49-4-1615 10555343

[mbo31200-bib-0013] Cook, S. J. , Graham, D. J. , Swift, D. A. , Midgley, N. G. , & Adam, W. G. (2011). Sedimentary signatures of basal ice formation and their preservation in ice‐marginal sediments. Geomorphology, 125, 122–131. 10.1016/j.geomorph.2010.08.018

[mbo31200-bib-0014] Cook, S. J. , Knight, P. G. , Waller, R. I. , Robinson, Z. P. , & Adam, W. G. (2007). The geography of basal ice and its relationship to glaciohydraulic supercooling: Svinafellsjokull, southeast Iceland. Quaternary Science Reviews, 26, 2309–2315.

[mbo31200-bib-0015] Cook, S. J. , Robinson, Z. P. , Fairchild, I. J. , Knight, P. G. , Waller, R. I. , & Boomer, I. (2010). Role of glaciohydraulic supercooling in the formation of stratified facies basal ice: Svínafellsjökull and Skaftafellsjökull, southeast Iceland. Boreas, 39, 24–38.

[mbo31200-bib-0016] Cook, S. J. , Swift, D. A. , Graham, D. J. , & Midgley, N. G. (2011). Origin and significance of “dispersed facies” basal ice: Svinafellsjokull, Iceland. Journal of Glaciology, 57, 710–720.

[mbo31200-bib-0017] Csardi, G. , & Csardi, M. G. (2007) The igraph package.

[mbo31200-bib-0018] Csardi, G. , & Nepusz, T. (2006). The igraph software package for complex network research. InterJournal, Complex Systems, 1695, 1–9.

[mbo31200-bib-0019] DeSantis, T. Z. , Hugenholtz, P. , Larsen, N. , Rojas, M. , Brodie, E. L. , Keller, K. , … Andersen, G. L. (2006). Greengenes, a Chimera‐Checked 16S rRNA Gene Database and Workbench Compatible with ARB. Applied and Environmental Microbiology, 72(7), 5069–5072. 10.1128/AEM.03006-05 16820507 PMC1489311

[mbo31200-bib-0020] Diao, M. , Sinnige, R. , Kalbitz, K. , Huisman, J. , & Muyzer, G. (2017). Succession of bacterial communities in a seasonally stratified lake with an anoxic and sulfidic hypolimnion. Frontiers in Microbiology, 8, 2511. 10.3389/fmicb.2017.02511 29312212 PMC5735980

[mbo31200-bib-0021] Dunham, E. C. , Dore, J. E. , Skidmore, M. L. , Roden, E. E. , & Boyd, E. S. (2021). Lithogenic hydrogen supports microbial primary production in subglacial and proglacial environments. Proceedings of the National Academy of Sciences of the United States of America, 118, e2007051117. 33419920 10.1073/pnas.2007051117 PMC7812807

[mbo31200-bib-0022] Ebert, K. T. (2003). Identifying glaciohydraulic supercooling at Hoffellsjèokull and Kviârjokull. .

[mbo31200-bib-0023] Edwards, A. , Douglas, B. , Anesio, A. M. , Rassner, S. M. , Irvine‐Fynn, T. D. L. , Sattler, B. , & Griffith, G. W. (2013). A distinctive fungal community inhabiting cryoconite holes on glaciers in Svalbard. Fungal Ecology, 6, 168–176. 10.1016/j.funeco.2012.11.001

[mbo31200-bib-0024] Edwards, A. , Rassner, S. M. E. , Anesio, A. M. , Worgan, H. J. , Irvine‐Fynn, T. D. L. , Wyn Williams, H. , … Wyn Griffith, G. (2013). Contrasts between the cryoconite and ice‐marginal bacterial communities of Svalbard glaciers. Polar Research, 32, 1–9. 10.3402/polar.v32i0.19468

[mbo31200-bib-0025] Elser, J. J. , Bastidas Navarro, M. , Corman, J. R. , Emick, H. , Kellom, M. , Laspoumaderes, C. , … Modenutti, B. (2015). Community structure and biogeochemical impacts of microbial life on floating pumice. Applied and Environmental Microbiology, 81, 1542–1549. 10.1128/AEM.03160-14 25527547 PMC4325165

[mbo31200-bib-0026] Fernández‐Martínez, M. A. , Pointing, S. B. , Pérez‐Ortega, S. , Arróniz‐Crespo, M. , Green, T. G. , Rozzi, R. (2016). Functional ecology of soil microbial communities along a glacier forefield in Tierra del Fuego (Chile). International Microbiology, 19, 161–173. 28494086 10.2436/20.1501.01.274

[mbo31200-bib-0027] Frey, B. , Rieder, S. R. , Brunner, I. , Plötze, M. , Koetzsch, S. , Lapanje, A. , … Furrer, G. (2010). Weathering‐Associated bacteria from the damma glacier forefield: Physiological capabilities and impact on granite dissolution. Applied and Environmental Microbiology, 76, 4788–4796. 10.1128/AEM.00657-10 20525872 PMC2901745

[mbo31200-bib-0028] Giraudoux, P. (2018). pgirmess: Spatial Analysis and Data Mining for Field Ecologists.

[mbo31200-bib-0029] Gomez, M. R. , Cerutti, S. , Sombra, L. L. , Silva, M. F. , & Martínez, L. D. (2007). Determination of heavy metals for the quality control in argentinian herbal medicines by ETAAS and ICP‐OES. Food and Chemical Toxicology, 45, 1060–1064. 10.1016/j.fct.2006.12.013 17291663

[mbo31200-bib-0030] Harrell, F. E. Jr . (2013) Hmisc: Harrell miscellaneous. R package version 3.12‐2. Computer software].

[mbo31200-bib-0031] Helgason, J. , & Duncan, R. A. (2001). Glacial‐interglacial history of the Skaftafell region, southeast Iceland, 0–5 Ma. Geology, 29, 179–182. 10.1130/0091-7613(2001)029<0179:GIHOTS>2.0.CO;2

[mbo31200-bib-0032] Hodson, A. , Anesio, A. M. , Tranter, M. , Fountain, A. , Osborn, M. , Priscu, J. , … Sattler, B. (2008). Glacial ecosystems. Ecological Monographs, 78, 41–67. 10.1890/07-0187.1

[mbo31200-bib-0035] Hotaling, S. , Hood, E. , & Hamilton, T. L. (2017). Microbial ecology of mountain glacier ecosystems: Biodiversity, ecological connections, and implications of a warming climate. Environmental Microbiology, 19(8), 2935–2948. 10.1111/1462-2920.13766 28419666

[mbo31200-bib-0036] Hubbard, B. , Cook, S. , & Coulson, H. (2009). Basal ice facies: A review and unifying approach. Quaternary Science Reviews, 28, 1956–1969. 10.1016/j.quascirev.2009.03.005

[mbo31200-bib-0037] Hubbard, B. , & Sharp, M. (1995). Basal ice facies and their formation in the western Alps. Arctic and Alpine Research, 301–310. 10.2307/1552023

[mbo31200-bib-0038] Ives, J. D. (2007) Skaftafell in Iceland: A Thousand Years of Change, Ormstunga.

[mbo31200-bib-0039] Jing, G. , Sun, Z. , Wang, H. , Gong, Y. , Huang, S. , Ning, K. , … Su, X. (2017). Parallel‐META 3: Comprehensive taxonomical and functional analysis platform for efficient comparison of microbial communities. Scientific Reports, 7. 10.1038/srep40371 PMC5227994 28079128

[mbo31200-bib-0040] Kaczmarek, Ł. , Jakubowska, N. , Celewicz‐Gołdyn, S. , & Zawierucha, K. (2016). The microorganisms of cryoconite holes (algae, Archaea, bacteria, cyanobacteria, fungi, and Protista): a review. Polar Record, 52, 176–203. 10.1017/S0032247415000637

[mbo31200-bib-0041] Kandler, K. , Benker, N. , Bundke, U. , Cuevas, E. , Ebert, M. , Knippertz, P. , … Weinbruch, S. (2007). Chemical composition and complex refractive index of Saharan Mineral Dust at Izaña, Tenerife (Spain) derived by electron microscopy. Atmospheric Environment, 41, 8058–8074. 10.1016/j.atmosenv.2007.06.047

[mbo31200-bib-0042] Kayani, M. U. R. , Doyle, S. M. , Sangwan, N. , Wang, G. , Gilbert, J. A. , Christner, B. C. , & Zhu, T. F. (2018). Metagenomic analysis of basal ice from an Alaskan glacier. Microbiome, 6, 123. 10.1186/s40168-018-0505-5 29976249 PMC6034282

[mbo31200-bib-0043] Knight, P. G. (1997). The basal ice layer of glaciers and ice sheets. Quaternary Science Reviews, 16, 975–993. 10.1016/S0277-3791(97)00033-4.

[mbo31200-bib-0044] Knight, P. G. , & Knight, D. A. (1994). Glacier sliding, regelation water flow and development of basal ice. Journal of Glaciology, 40, 600–601. 10.1017/S0022143000012491

[mbo31200-bib-0045] Kozich, J. J. , Westcott, S. L. , Baxter, N. T. , Highlander, S. K. , & Schloss, P. D. (2013). Development of a dual‐index sequencing strategy and curation pipeline for analyzing amplicon sequence data on the MiSeq Illumina sequencing platform. Applied and Environmental Microbiology, 79, 5112–5120. 10.1128/AEM.01043-13 23793624 PMC3753973

[mbo31200-bib-0046] Langille, M. G. I. , Zaneveld, J. , Caporaso, J. G. , McDonald, D. , Knights, D. , Reyes, J. A. , … Huttenhower, C. (2013). Predictive functional profiling of microbial communities using 16S rRNA marker gene sequences. Nature Biotechnology, 31, 814–821. 10.1038/nbt.2676 PMC3819121 23975157

[mbo31200-bib-0047] Laskin, A. , Cowin, J. P. , & Iedema, M. J. (2006). Analysis of individual environmental particles using modern methods of electron microscopy and X‐ray microanalysis. Journal of Electron Spectroscopy and Related Phenomena, 150, 260–274. 10.1016/j.elspec.2005.06.008

[mbo31200-bib-0048] Lawson, E. C. , Wadham, J. L. , Lis, G. P. , Tranter, M. , Pickard, A. E. , Stibal, M. (2015). Identification and analysis of low molecular weight dissolved organic carbon in subglacial basal ice ecosystems by ion chromatography. Biogeosciences Discussions, 12, 14139–14174.

[mbo31200-bib-0049] Lutz, S. , Anesio, A. M. , Edwards, A. , & Benning, L. G. (2015). Microbial diversity on Icelandic glaciers and ice caps. Frontiers in Microbiology, 6, 10.3389/fmicb.2015.00307 PMC4403510 25941518

[mbo31200-bib-0050] Lutz, S. , Anesio, A. M. , Jorge Villar, S. E. , & Benning, L. G. (2014). Variations of algal communities cause darkening of a Greenland glacier. FEMS Microbiology Ecology, 89, 402–414. 10.1111/1574-6941.12351 24920320

[mbo31200-bib-0051] Macey, M. C. , Fox‐Powell, M. , Ramkissoon, N. K. , Stephens, B. P. , Barton, T. , Schwenzer, S. P. (2020). The identification of sulfide oxidation as a potential metabolism driving primary production on late Noachian Mars. Scientific Reports, 10, 10941. 32616785 10.1038/s41598-020-67815-8 PMC7331718

[mbo31200-bib-0052] Macreadie, P. I. , Allen, K. , Kelaher, B. P. , Ralph, P. J. , & Skilbeck, C. G. (2012). Paleoreconstruction of estuarine sediments reveal human‐induced weakening of coastal carbon sinks. Global Change Biology, 18, 891–901. 10.1111/j.1365-2486.2011.02582.x

[mbo31200-bib-0053] Madigan, M. , Martinko, J. , Stahl, D. , & Clark, D. (2010). Brock Biology of Microorganisms, 13th, ed. .

[mbo31200-bib-0054] McMahon, S. , Parnell, J. , & Blamey, N. J. F. (2016). Evidence for seismogenic hydrogen gas, a potential microbial energy source on Earth and Mars. Astrobiology, 16, 690–702. 10.1089/ast.2015.1405 27623198

[mbo31200-bib-0055] Mitchell, A. C. , Lafrenière, M. J. , Skidmore, M. L. , & Boyd, E. S. (2013). Influence of bedrock mineral composition on microbial diversity in a subglacial environment. Geology, 41, 855–858. 10.1130/G34194.1

[mbo31200-bib-0056] Montross, S. , Skidmore, M. , Christner, B. , Samyn, D. , Tison, J.‐L. , Lorrain, R. , … Fitzsimons, S. (2014). Debris‐Rich Basal Ice as a Microbial Habitat, Taylor Glacier, Antarctica. Geomicrobiology Journal, 31, 76–81. 10.1080/01490451.2013.811316

[mbo31200-bib-0057] Mustakhimov, I. , Kalyuzhnaya, M. G. , Lidstrom, M. E. , & Chistoserdova, L. (2013). Insights into denitrification in Methylotenera mobilis from denitrification pathway and methanol metabolism mutants. Journal of Bacteriology, 195, 2207–2211. 10.1128/JB.00069-13 23475964 PMC3650538

[mbo31200-bib-0058] Rime, T. , Hartmann, M. , & Frey, B. (2016). Potential sources of microbial colonizers in an initial soil ecosystem after retreat of an alpine glacier. The ISME Journal. 10.1038/ismej.2015.238 PMC4918445 26771926

[mbo31200-bib-0059] Santelli, R. E. , Oliveira, E. P. , de Carvalho, M. D. F. B. , Bezerra, M. A. , & Freire, A. S. (2008). Total sulfur determination in gasoline, kerosene and diesel fuel using inductively coupled plasma optical emission spectrometry after direct sample introduction as detergent emulsions. Spectrochimica Acta Part B: Atomic Spectroscopy, 63, 800–804. 10.1016/j.sab.2008.04.020

[mbo31200-bib-0060] Seewald, J. S. (2017) Detecting molecular hydrogen on Enceladus. Science, 356(6334), 132–133. 10.1126/science.aan0444 28408557

[mbo31200-bib-0061] Stevens, T. O. , & McKinley, J. P. (2000). Abiotic controls on H2 production from Basalt‐ Water reactions and implications for aquifer biogeochemistry. Environmental Science & Technology, 34, 826–831. 10.1021/es0015996 11348102

[mbo31200-bib-0062] Stibal, M. , GÃ¶zdereliler, E. , Cameron, K. A. , Box, J. E. , Stevens, I. T. , Gokul, J. K. , … Jacobsen, C. S. (2015). Microbial abundance in surface ice on the Greenland Ice Sheet. Frontiers in Microbiology, 6, 225. 10.3389/fmicb.2015.00225 25852678 PMC4371753

[mbo31200-bib-0063] Stibal, M. , Hasan, F. , Wadham, J. L. , Sharp, M. J. , & Anesio, A. M. (2012). Prokaryotic diversity in sediments beneath two polar glaciers with contrasting organic carbon substrates. Extremophiles, 16, 255–265. 10.1007/s00792-011-0426-8 22241643

[mbo31200-bib-0064] Stibal, M. , Wadham, J. L. , Lis, G. P. , Telling, J. , Pancost, R. D. , Dubnick, A. , … Anesio, A. M. (2012). Methanogenic potential of Arctic and Antarctic subglacial environments with contrasting organic carbon sources. Global Change Biology, 18, 3332–3345. 10.1111/j.1365-2486.2012.02763.x

[mbo31200-bib-0065] Swift, D. A. , Cook, S. J. , Graham, D. J. , Midgley, N. G. , Fallick, A. E. , Storrar, R. , … Evans, D. (2018). Terminal zone glacial sediment transfer at a temperate overdeepened glacier system. Quaternary Science Reviews, 180, 111–131. 10.1016/j.quascirev.2017.11.027

[mbo31200-bib-0066] Swift, D. A. , Midgley, N. G. , Graham, D. J. , Fallick, A. E. , Evans, D. J. A. , & Cook, S. J. (2006). Structural glaciology and debris transport at temperate glaciers with terminal overdeepenings: the examples of Kvíárjökull and Svínafellsjökull southeast Iceland. Geophysical Research Abstracts, 7123.

[mbo31200-bib-0067] Telling, J. , Boyd, E. S. , Bone, N. , Jones, E. L. , Tranter, M. , MacFarlane, J. W. , … Hodgson, D. A. (2015). Rock comminution as a source of hydrogen for subglacial ecosystems. Nature Geoscience, 8, 851–855. 10.1038/ngeo2533

[mbo31200-bib-0068] Temkiv, T. Š. , Finster, K. , Hansen, B. M. , Nielsen, N. W. , & Karlson, U. G. (2012). The microbial diversity of a storm cloud as assessed by hailstones. FEMS Microbiology Ecology, 81, 684–695. 10.1111/j.1574-6941.2012.01402.x 22537388

[mbo31200-bib-0069] Toubes‐Rodrigo, M. , Cook, S. J. , Elliott, D. , & Sen, R. (2016). In Geomorphological tehcniques. S. J. Cook , L. E. Clarke , & J. M. Nield (Ed.), Sampling and describing glacier ice, Vol. 3 (p. 1). British Society for Geomorphology.

[mbo31200-bib-0070] Toubes‐Rodrigo, M. , Cook, S. J. , Elliott, D. , & Sen, R. (2016). Quantification of basal ice microbial cell delivery to the glacier margin. Biogeosciences Discussions, 1–8.

[mbo31200-bib-0071] Valdivia‐Silva, J. E. , Navarro‐González, R. , Fletcher, L. , Perez‐Montaño, S. , Condori‐Apaza, R. , & Mckay, C. P. (2012). Soil carbon distribution and site characteristics in hyper‐arid soils of the Atacama Desert: a site with Mars‐like soils. Advances in Space Research, 50, 108–122. 10.1016/j.asr.2012.03.003

[mbo31200-bib-0072] Vilmundardóttir, O. K. , Gísladóttir, G. , & Lal, R. (2015). Soil carbon accretion along an age chronosequence formed by the retreat of the Skaftafellsjökull glacier, SE‐Iceland. Geomorphology, 228, 124–133. 10.1016/j.geomorph.2014.08.030

[mbo31200-bib-0073] Wang, K. , Ye, X. , Zhang, H. , Chen, H. , Zhang, D. , & Liu, L. (2016). Regional variations in the diversity and predicted metabolic potential of benthic prokaryotes in coastal northern Zhejiang, East China Sea. Scientific Reports, 6, 38709. 10.1038/srep38709 27917954 PMC5137025

[mbo31200-bib-0074] Wickham, H. (2009). ggplot2: Elegant Graphics for Data Analysis.

[mbo31200-bib-0075] Williams, M. , Villarreal, A. , Bozhilov, K. , Lin, S. , & Talbot, P. (2013). Metal and silicate particles including nanoparticles are present in electronic cigarette cartomizer fluid and aerosol. PLoS One, 8, e57987. 10.1371/journal.pone.0057987 23526962 PMC3603976

[mbo31200-bib-0076] Womack, A. M. , Bohannan, B. J. M. , & Green, J. L. (2010). Biodiversity and biogeography of the atmosphere. Philosophical Transactions of the Royal Society of London B: Biological Sciences, 365, 3645–3653. 10.1098/rstb.2010.0283 20980313 PMC2982008

[mbo31200-bib-0077] Yde, J. C. , Finster, K. W. , Raiswell, R. , Steffensen, J. P. , Heinemeier, J. , Olsen, J. , … Nielsen, O. B. (2010). Basal ice microbiology at the margin of the Greenland ice sheet. Annals of Glaciology, 51, 71–79. 10.3189/172756411795931976

